# Process elements contributing to community mobilization for HIV risk reduction and gender equality in rural South Africa

**DOI:** 10.1371/journal.pone.0225694

**Published:** 2019-12-02

**Authors:** Catherine MacPhail, Nomhle Khoza, Sarah Treves-Kagan, Amanda Selin, Xavier Gómez-Olivé, Dean Peacock, Dumisani Rebombo, Rhian Twine, Suzanne Maman, Kathleen Kahn, Stephanie M. DeLong, Lauren M. Hill, Sheri A. Lippman, Audrey Pettifor

**Affiliations:** 1 School of Health and Society, University of Wollongong, Wollongong, New South Wales, Australia; 2 Wits Reproductive Health & HIV Institute, School of Clinical Medicine, Faculty of Health Sciences, University of the Witwatersrand, Johannesburg, South Africa; 3 MRC/Wits Rural Public Health and Health Transitions Research Unit (Agincourt), School of Public Health, Faculty of Health Sciences, University of the Witwatersrand, Johannesburg, South Africa; 4 STRIVE Research Programme Consortium, London School of Hygiene and Tropical Medicine, London, England, United Kingdom; 5 Center for AIDS Prevention Studies (CAPS), Department of Medicine, University of California, San Francisco, California, United States of America; 6 Carolina Population Centre, University of North Carolina at Chapel Hill, Chapel Hill, North Carolina, United States of America; 7 INDEPTH Network, Accra, Ghana; 8 Sonke Gender Justice, Cape Town, South Africa; 9 Department of Health Behaviour, University of North Carolina Gillings School of Global Public Health, Chapel Hill, North Carolina, United States of America; 10 Epidemiology and Global Health Unit, Department of Public Health and Clinical Medicine, Umeå University, Umeå, Sweden; 11 Department of Epidemiology, University of North Carolina Gillings School of Global Public Health, Chapel Hill, North Carolina, United States of America; University of South Florida, UNITED STATES

## Abstract

Community mobilization has been recognized as a critical enabler for HIV prevention and is employed for challenging gender inequalities. We worked together with community partners to implement the ‘One Man Can’ intervention in rural Mpumalanga, South Africa to promote gender equality and HIV risk reduction. During the intervention, we conducted longitudinal qualitative interviews and focus group discussions with community mobilizers (n = 26), volunteer community action team members (n = 22) and community members (n = 52) to explore their experience of being part of the intervention and their experiences of change associated with the intervention. The objective of the study was to examine processes of change in community mobilization for gender equity and HIV prevention. Our analysis showed that over time, participants referred to three key elements of their engagement with the intervention: developing respect for others; inter-personal communication; and empathy. These elements were viewed as assisting them in adopting a ‘better life’ and associated with behaviour change in the intervention’s main focus areas of promoting gender equality and HIV risk reduction behaviours. We discuss how these concepts relate to the essential domains contained within our theoretical framework of community mobilization—specifically critical consciousness, shared concerns and social cohesion -, as demonstrated in this community. We interpret the focus on these key elements as significant indicators of communities engaging with the community mobilization process and initiating movement towards structural changes for HIV prevention.

## Introduction

Strength, control, and sexual prowess dominate constructions of the masculine ideal in South African society. These defining features of masculine identity are enacted through a range of sexual risk practices as well as the domination of and use of violence against women [1–3]. Subscribing to these masculine ideals has a significant impact on the health of both men and women, being associated with substance use, transactional sex, infrequent condom use and poor utilisation of health care services, including HIV testing and care [1, 4–10] Such behaviours constitute the backdrop to South Africa’s twin epidemics of HIV and intimate partner violence [11, 12] with national HIV prevalence among South Africans aged 15–49 estimated at 14.8% and 26.3% for men and women respectively [13] and physical violence towards a partner reported among 30–50 percent of men [14].

In HIV endemic countries, there is increasing recognition of community mobilization as a ‘critical enabler’ for appropriate HIV responses [15, 16]. In this context, community mobilization is viewed as the process to engage community members in working towards a common goal. HIV prevention through community mobilization builds on an understanding of HIV risk behaviour as being driven by community and social norms and structural drivers, including normative gender roles and behaviours [17–19]. Community mobilization has had some success in challenging the social factors that place people at risk for HIV, and has impacted behavioural outcomes such as increasing uptake of HIV testing, consistent condom use and utilisation of STI testing and treatment [20–26]. Despite these successes, there is a need to better understand the processes and mechanisms of community mobilization in order to refine this approach and enhance future programming.

In an attempt to promote further understanding of how a community mobilization process occurs, we conducted longitudinal qualitative data collection as part of a cluster randomized-controlled trial of a community mobilization intervention based on a theory of Community Mobilisation (CM) [27], which aimed to modify inequitable gender norms and decrease HIV risk behaviours. From a review of literature on social movements, community empowerment, community development and community capacity conducted prior to the intervention, we established six key domains in which change is deemed necessary for effective community mobilization to occur. The six domains are 1) shared concern; 2) community consciousness; 3) organizational structure/networks; 4) leadership; 5) collective actions; and 6) social cohesion [27]. These domains informed the design and content of intervention activities and our evaluation interpretation.

### The one man can intervention

Based on the theoretical domains, we designed and implemented a community-based intervention in 11 villages randomly selected from 22 villages within the HDSS; the intervention was evaluated through a community randomized trial. The intervention was co-developed and delivered by Sonke Gender Justice, a South African non-profit organisation. Sonke Gender Justice works with communities throughout Africa, specifically using advocacy and community mobilisation to focus on violence prevention, gender equity and child rights [28]. The intervention targeted men and women aged 18–35 years and was adapted from Sonke Gender Justice’s One Man Can (OMC) campaign [29] and focused on increasing awareness of the relationship between gender inequalities and HIV. Our adaptation of OMC specifically expanded a focus on the intersection between HIV and gender, and therefore focused on seven thematic areas: gender, power and health; gender and violence; gender and HIV/AIDS; alcohol; healthy relationships; human rights; and, taking action. These thematic areas map onto the theoretical framework previously mentioned [27].

A single individual (or two in larger villages) in each intervention village was recruited to act as a community mobiliser, to lead community mobilization activities and assist in establishing and training volunteer Community Action Teams (CATs). Community-based activities included intensive small group workshops, informal community dialogue through door-to-door home visits, mural painting to stimulate discussions of key messages, informal theatre and discussion, soccer tournaments and film/digital stories. These were conducted in a range of community venues such as church halls, community halls, taverns and informal meeting places such as taxi ranks and outside shops [30]. The intervention ran from July 2012 to July 2014. During this time 37.3% of 18–35 year old men were reached with at least one 2-day workshop and just under 6,000 activities were conducted in the intervention communities [31].

Quantitative evaluation of the intervention was premised on the Gender Equitable Men’s Scale (GEMS) as the main outcome, with secondary outcomes including reported multiple sex partners in the past 12 months; condom use at last sex; experience/perpetration of intimate partner violence (IPV) and harmful use of alcohol in the past 12 months [32]. At the conclusion of the intervention, we found a significant increase in GEMS score (signifying more equitable gender attitudes) among intervention community men when compared to control men (Beta coefficient 95% CI: 0.62; 4.78). No significant results were reported for women, although both reports of multiple partners and experience of IPV declined among women in intervention communities [31].

If community mobilization is to be encouraged as a critical enabler of HIV interventions, we need to better understand both the constituent parts of community mobilization, but also the steps through which communities move as they mobilize around specific issues. This paper qualitatively explores community and intervention staff perceptions of the change process of individuals and communities, using data drawn from longitudinal qualitative interviews.

## Materials and methods

### Setting

This research is situated in the rural Bushbuckridge sub-district, Mpumalanga Province of South Africa, in the Agincourt Health and Demographic Surveillance System (Agincourt HDSS) site. The area is located about 500kms northeast of Johannesburg, close to the border with Mozambique. It is characterized by high levels of unemployment, significant temporary migration and high HIV prevalence:19.4 per cent among those older than 15 years in 2010–2011, and peak prevalence of more than 40 percent [33].

### Selection of participants

For the qualitative study, four intervention villages were purposively selected in order to account for diversity of initial community engagement in mobilization [34], from straightforward to demanding, in consultation with OMC staff. Villages ranged significantly in size and all had young populations (broadly representative of the Agincourt sub-district as a whole) (Table 1).

**Table 1:** Villages included in the qualitative sub-study.

	Village A	Village B	Village C	Village D
**Population**	6 243	6 396	2 095	865
**Experience of implementing OMC**	Average	Average	Easy	Challenging

During 2012, OMC intervention staff identified individuals attending the first community workshops in the 11 selected villages. These men and women were invited to enrol in longitudinal, qualitative interviews [35] after the purpose of the study was discussed. We purposively selected five participants per village, three men (n = 12) and two women (n = 8) and focused on individuals who OMC staff had identified as participants who shared their thoughts freely during initial intervention workshops. These individuals were considered likely to be good sources of information about their communities. Additionally, we invited all community mobilisers (n = 17) and CATs to participate in interviews and focus group discussions (FGDs), respectively. Data collection was conducted from October 2012 to May 2014.

### Interviews and FGDs

Community members were interviewed three times, at 6, 12 and 18 months into the intervention. The community mobilisers (staff of OMC) were interviewed at two time points and CATs (one CAT in each of 11 villages) participated in FGDs twice, both at 6 and 18 months. Participants provided written consent in the first interview and were verbally re-consented at each subsequent interview. A team of four female qualitatively-trained, XiTsonga speaking fieldworkers with high school qualifications completed data collection alone with each participant or group. Each fieldworker was allocated specific participants for relational continuity over the duration of the study. Interviews took approximately 1–2 hours and were conducted at participants’ homes or other locations of their choosing. FGDs were conducted in local schools.

The interview and FGD guides (see S1, S2 and S3 Appendices) explored experiences with the intervention, reflections on personal change, perceptions of community change, and understanding of village community mobilization processes. At each follow-up interview/FGD participants were asked to discuss events and experiences since the last time they had been interviewed and whether there had been changes in their experiences of the intervention, mobilization or personal/community attitudes towards HIV or gender equity since their last interview. Interviews and FGDs were digitally recorded and then translated and transcribed into English by the same interviewer who conducted each interview. Summary notes were also taken during the interview in case of recording failure.

### Analysis

Transcripts were checked for accuracy and completeness by NK, who read through each transcript and returned to the original recording for clarity where information did not make sense or was documented as not being audible. The transcript was returned to the interviewer to clarify and correct. Transcripts were then imported into Atlas.ti for coding and identification of dominant themes using a framework analysis approach [36] in which the research team works through five main analytical steps. The steps include: familiarisation with the raw data; identifying a thematic framework that draws of a priori issues and questions; indexing; charting; and mapping and interpretation of the data. The code frame was developed by two of the study team (CM and NK), with further codes added inductively as the first interviews were coded. The final code frame included 107 codes across ten main thematic areas. Coding was completed by two members of the study team (NK and ST-K) after achieving adequate intercoder reliability with kappa values of above 0.70 during initial coding of the first ten transcripts [37]. Intercoder reliability was checked throughout the analysis by double-coding every tenth transcript.

We conducted analysis to capture processes and experiences over the duration of the intervention [38]. Theme areas were allocated to an individual member of the team for analysis but we collectively discussed our findings and interpretations of the data at multiple points. Team members summarized each participant’s or CAT’s transcripts by creating analytic matrices with participants on the x-axis and time on the y-axis (both at the individual level and reported community level). Thereafter further matrices were constructed to consolidate patterns for each thematic area (main study outcomes) and for each type of participant included in the analysis (i.e. community member, mobiliser or CAT). Given that we were most interested in the process of changes associated with participation in the community mobilization, we limited our analysis to data from individuals who had completed at least one follow-up interview.

Study processes were conducted according to the COREQ checklist for qualitative studies [39] (S4 Appendix). The study was approved by the Institutional Review Board of the University of North Carolina at Chapel Hill, the Human Research Ethics Committee of the University of the Witwatersrand and the Mpumalanga Departments of Health and Social Development Research and Ethics Committees.

## Results

Our final sample included community mobilisers (13 of 17), community members (19 of 25) and all 11 CATs across 78 individual interviews (52 with community members) and 22 FGDs (see Table 2). Quotes included in the paper originate from 9, 9 and 13 CATS, community mobilisers and community members respectively. During data collection, five participants chose to withdraw from participation due to increased work commitments and were replaced by a sex-matched individual from the same village. This explains the additional community members beyond the original 20 recruited for the study. There was no difference by sex or age in terms of participation in data collection, although community participants from village B were less likely to have completed all three interviews than participants from other villages. Community members ranged in age from 20 to 44 years and included almost equal numbers of men (n = 10) and women (n = 9). There were five community participants each from villages A, C and D; and four participants from village B. Community mobilisers were largely aged 18–25 years, equally distributed by sex and represented 10 of the 11 intervention villages. The number of individuals participating in each of the CAT FGDs differed between villages and time points; a minimum of three and maximum ten participants. Participants were largely within the intervention target age range (18–35 years), although we did not limit participation by age.

**Table 2:** Participant characteristics.

Individual	Sex	Age	Village	No. of Interviews
**Community Members (52 interviews with 19 individuals)**				
Community member 1	Male	18–35 years	Village C	3
Community member 2	Male	18–35 years	Village C	3
Community member 3	Female	18–35 years	Village C	3
Community member 4	Female	18–35 years	Village C	3
Community member 5	Male	18–35 years	Village A	3
Community member 6	Male	No date	Village A	3
Community member 7	Female	18–35 years	Village A	3
Community member 8	Female	>35 years	Village A	2
Community member 9	Female	18–35 years	Village B	2
Community member 10	Female	18–35 years	Village B	2
Community member 12	Male	18–35 years	Village B	2
Community member 13	Male	No data	Village A	2
Community member 14	Male	No data	Village B	3
Community member 15	Male	>35 years	Village D	3
Community member 16	Male	>35 years	Village D	3
Community member 17	Female	18–35 years	Village D	3
Community member 18	Female	18–35 years	Village D	3
Community member 19	Male	18–35 years	Village D	3
Community member 20	Female	18–35 years	Village C	3
**Community Mobilizers (26 interviews with 13 individuals)**				
Mobilizer 2	Male	18–35 years	Village A	2
Mobilizer 3	Female	18–35 years	Village A	2
Mobilizer 4	Female	18–35 years		2
Mobilizer 5	Female	No data		2
Mobilizer 6	Male	18–35 years	Village C	2
Mobilizer 7	Male	18–35 years		2
Mobilizer 8	Female	18–35 years	Village B	2
Mobilizer 9	Male	18–35 years		2
Mobilizer 10	Female	>35 years	Village D	2
Mobilizer 11	Male	18–35 years		2
Mobilizer 12	Male	18–35 years		2
Mobilizer 13	Female	18–35 years		2
Mobilizer 14	Female	>35 years		2

Our analysis of the participants’ reflections on their engagement with the intervention pointed to three significant thematic areas relating to gender equitable transformation: respect, communication, and empathy. The participants made frequent reference to these elements as being significant for a ‘better life’ in their communities; they associated them with the OMC intervention and with their attempts to change behaviours related to the intervention outcomes. We focus our discussion on these elements before making remarks about the process of change more generally in the context of this intervention.

### Respect

Respect was a key element that emerged from our analysis of the process of change in attitudes and reported behaviours. Participants particularly noted that they needed to learn to respect themselves as a precursor to respecting others and for mutually beneficial, safe relationships. For example, a local business man noted that a key message from OMC was that ‘if I respect myself, you won’t disrespect me’ (Community member 13, male). Respect was most commonly discussed when participants reflected on their experiences in the context of gender equality and sexual behaviour change, although it was also discussed as a key element of the program’s focus on the link between alcohol misuse and violence, poor domestic relationships and risky sexual behaviours.

In one of the CAT focus group discussions, a male member of the team acknowledged that increased respect was at the core of changes in attitudes about women’s roles and their responsibilities within the household:

With gender equality, we as the males, we were hiding ourselves and we didn’t know how to live with our wives. But now it’s interesting that males are learning that females deserve to be respected and also they need assistance in every household chore.

(Community Advisory Committee 9, 6 month interview)

This was echoed in other discussion groups with CATs where the participants spoke of having a greater appreciation for the various roles that women performed in the household:

Yes I have noticed the change that has occurred on gender; the boys who attended the workshop of OMC are now able to clean their household‘s yard and washes the dishes. They consider the females more and they are doing this as a way of showing that they are honouring us.

(Community Advisory Committee 1, 12 month interview)

The relationship between respect and behaviour change was also discussed in the individual interviews. For example, key to the reflections on gender equality of a female community mobiliser was the notion that respect facilitates deeper consideration of potential future alternatives to the current status quo:

I know that gender equality goes together with love and respect. If you respect your partner you will have sympathy in everything, not that you do the same job. You will live a better life in your household because you will communicate with each other. This will make your relationship easier because of love and respect.

(Community mobilizer 3, female, 18–35 years. 6 month interview)

For the male community mobilisers, their reflections centred on the manner in which they had learned to focus on and show respect, specifically in terms of gender equality. For example, a 20-year-old male mobiliser discussed his increased respect for women when he noted:

… I treat a woman as a human being… eeh … I am no longer treating her [just]as a woman but I treat her as a human being. The way I want someone to treat me.

(Community mobilizer 9; male, 18–35 years, 18 month interview)

His description of personal change was echoed by another male mobiliser in his early twenties:

Aaah, like I was involved in relationships with many girls and drinking alcohol. I was causing problems like violence and I was not considering other people’s opinion. I use to tell people that I am ‘a boss’ and I disrespected people… but since I’m involved [in OMC] I am able to know who I am, why I’m here in the world, what I want in this world.

(Community mobilizer 6, male, 18–35 years, 6 month interview)

It was apparent that among community members, both men and women struggled to recognize that gender equality is not synonymous with taking respect away from men. Their comments about increased respect for women were often tempered by admonishments to maintain respect for men. A male community member (Community member 6, male, no age data, 12 month interview) noted ‘No I don’t have a problem with gender equality as long as we respect each other.’ This sentiment was also reflected in the comment by a female community member that ‘Gender equality is good because we are all equal. But we must not disrespect men’ (Community member 4, female, 18–35 years, 6 month interview).

A number of male community members particularly raised concerns about implementing gender equality in the home. For example, a male community member was enthusiastic about the changes he saw as a result of OMC, yet struggled with accepting gender equality in his own home. His comment indicates that he still needed to be perceived as head of his household and interpreted gender inequality in those terms: ‘Gender equality is working in my house because I don’t put my wife under pressure, and she’s giving me the respect that I deserve’ (Community member 14, male, no age data, 18 month interview). These ambivalence around the distribution and redistribution of power occurred in the comments of a minority of participants and should be contrasted with other comments emphasizing that women’s equality did not mean loss of respect for men, as illustrated by the following community member who remarked that ‘Even women have rights to make decisions, but they are still respecting their men’ (Community member 2, male, 18–35 years, 12 month interview).

The notion of respect was also discussed more broadly as an inherent component of leading a better or ‘good’ life. In one of the CAT discussions, a participant mentioned that he had noticed ‘people are now respecting their lives.’ (Community Action Team 9, 6 month interview)

Others also expanded the notion of respect from being only about gender equality to a requirement in their communities more broadly. One of the older men interviewed from the community highlighted this issue when he stated that ‘… the most important thing in the family is to respect each other’ (Community member 15, male, 12 month interview).

### Communication

Communication was another key element nominated by our participants as key to the process of making change in gender equality, HIV testing behaviours, sexual behaviour and violence. As one female participant noted:

The time of keeping things quiet, even if it is not good, has passed now. One Man Can is encouraging people to share their experience or problems.

(Community member 8, female, >35 years, 6 month interview)

The importance of communication was discussed as impacting on better relationships, within families as well as sexual partnerships. A female community mobiliser who lives with her husband and two children spoke about using the information that she learned through OMC in her family. She enthusiastically commented:

There is a great change with me, even my husband says that One Man Can made changes in our lives. It teaches me to communicate well with my husband and now we know about our health status [have tested for HIV]. And on how I should talk to my children.

(Community mobilizer 3, female, 18–35 years, 6 month interview)

This was echoed by others. One of the mobilisers reported experiencing conflict with her extended family; however, a focus on appropriate communication had assisted in managing both the conflict and resultant aggression:

Usually when there is a problem she [sister in law] becomes aggressive. She is unable to communicate well when there is a problem; she always behaves in a violent way. So now I know how to cool her down. If there is a problem, then we come up with the solution besides shouting at each other.

(Community mobilizer 4, female, 18–35 years, 18 month interview)

Similarly, a male teacher from the community discussed how he had embraced communication. He noted:

It [OMC] has transformed me … that I must understand that family is all about communication … Once you beat someone else it means you are failing to communicate and to convince.

(Community member 15, male, > 35 years, 12 month interview)

Communication was cited as being a key component to shifts in gender norms, sexual behaviour change and adoption of HIV prevention strategies. A CAT member (Community Action Team 2, 6 month interview) stated that ‘when it comes to gender there is communication in the house and no one who uses power’, while one of the male community members (Community member 6, male, no age data,) explained that ‘it doesn’t mean that when you are the man in a house you have to take the final decision, … but to sit down and talk; you can build a good thing.’

A mobiliser mentioned the role of communication in terms of condom use:

In the past, people were fighting if the partner came with the condom or maybe to use a condom was letting them down. But now we are communicating about it. Even myself, I use it. I simply communicate with my partner when we use it.

(Community mobilizer 4, female, 18–35 years, 6 month interview)

Across the interviews, a number of participants noted communication skills as essential to disclosing HIV status within their sexual relationships:

I now have one partner. I know her status and she knows my status. There is nothing we are scared of when it comes to HIV, as we speak.

(Community mobilizer 9, male, 18–35 years, 18 month interview)

Among community members, participants also commented on the benefits of increased communication. A woman living with her husband and extended family (Community member 7, female, 18–35 years, 6 month interview) explained ‘I am able to protect myself when having sex after I communicated with my partner in a good way. We don’t shout at each other. In everything we talk about we have a solution.’

Communication played a significant role in change at the community level. One of the community members (Community member 14, male, no age data, 6 month interview) discussed marked change in communication with regard to HIV. In his first interview, he noted that:

Our community seems like they are very ignorant when it comes to the issue of HIV. When we have a community meeting … I have never heard a community member raise a concern in particular about HIV, on how we can reduce it.

In contrast, in his third interview he stated that:

They are always discussing it. If I find myself in a group of people, there is never a time that they don’t discuss HIV (18 month interview).

Community-wide communication was discussed as mediating gender-based violence and addressing gender inequalities. A prominent community member, with significant roles in the local council and church, commented that men were talking about gender more than in the past:

Sometimes we have meeting of men in our community where we teach each other about how to behave as men in our community and what to do, [other] than causing violence, if you are in a relationship and you feel like you don’t want to continue with it.

(Community member 15, male, >35 years, 12 month interview)

Overall, engagement with OMC particularly facilitated the use of communication within households and families as a strategy to reduce violence, both in intimate and parent-child relationships.

### Empathy

Empathy was the third key underlying concept that emerged from the analysis of discussions about the impact of OMC with regard to gender equality and violence. Commenting on the changes she perceived to have taken place in her household, a community member explained how she now perceived men and women as more similar than they are different. In noting that ‘pain is the same and even blood runs in the same veins’ (Community member 3, female, 18–35 years, 6 month interview) she highlights empathetic regard for the other gender. Expressions of empathy were evident in the ways participants talked about others, both as individuals and for their role in society. For example, increased empathy for women encouraged more gender equitable attitudes and behaviours amongst the male participants, particularly evident in sharing domestic responsibilities and child care. For example, a CAT member spoke about how he now appreciated and assisted his mother with the domestic roles she performed:

I was unable to feel sorry for someone like… my mother, when she goes out to fetch water or firewood I was not having a problem. I thought it’s her duty to do it, but since I have got an experience from One Man Can I’m now able to assist where necessary

(Community Action Team 9, male, 6 month interview)

Changing attitudes were further reflected in comments from female CAT members in different communities:

When I was growing up I didn’t know that my brother can help me with other household chores like cooking or cleaning. So One Man Can helped me, because even men can do things that were supposed to be done by women. We grew up knowing that men have to find job and work for their families and women do household chores. So in One Man Can we learn that men can do things that should be done by women.

(Community Action Team 4, 6 month interview)

However, this empathy went beyond the burdens associated with women’s domestic responsibilities and was reflected in multiple statements in which participants described how men ‘now understand that a woman is a human being and she has feelings’ (Community member 15, male, >35 years, 18 month interview). Such empathetic understanding was particularly discussed with regards to violence against women: ‘I have learned that if you do violence to someone you must also think how that person will feel and how you would feel if they did it to you’ (Community Action Team 9, 18 month interview). A CAT member spoke of personal experience of learning empathy with regard to a family member’s experience of gender violence:

I remember one of my siblings got raped. After that, we were saying whatever we want to say, but we were unaware that she is feeling pain when we talk like that. But since I have started to attend One Man Can workshops I have learned that if you come across a bad situation it doesn’t mean that you have hunted for it [asked for it].

(Community Action Team 3, 6 month interview)

Similarly, a mobiliser recognized that in situations such as rape, acknowledging the victim’s feelings was as significant as dealing with the perpetrator. She explained:

Let’s take it someone has been raped by a family member then they agree to treat the issue as family and forget about the one who was raped and how that person feels. So now people are aware that it’s not good to hide something which is affecting someone’s life.

(Community mobilizer 4, female, 18–35 years, 18 month interview)

Women in particular discussed reducing physical violence against their children, as a result of now understanding more about the consequences for their children, with one female CAT member saying, ‘I didn’t know that if I beat my child its abuse and I hurt the child. That is what I learned. We are violent to our children not knowing that they will be affected when they grow up’ (Community Action Team 4, 6 month interview). Men who were particularly involved in the OMC intervention also discussed reducing violence towards others in the community as a consequence of growing empathy. For example, a CAT member reflected on how violence used to make him feel, and how he has since altered his behaviour, after developing some understanding of how his violence affected others:

I was feeling like ‘a boss’ if I have beaten someone, but since I started to participate in OMC I have noticed that it is not good, because when you fight someone you make the other person feel pain.

(Community Action Team 3; male, 6 month interview)

An understanding of how others might suffer pain as a consequence of actions that were initially taken for granted was also evident in relation to potential consequences of multiple partnerships. For example, in the following quote, a young male mobiliser reflects on his recognition of how multiple sexual partnerships have emotional consequences for the women involved and his choice to change his behaviour:

So, since I joined OMC and saw the risks of having multiple partners and also understand the pain that is there … the pain, there is no pain in a relationship that is painful like when a person cheats on you. So, I have changed there and have one girlfriend.

(Community mobilizer 2, male, 18–35 years, 18 month interview)

## Discussion

From this qualitative analysis of interviews and FGDs with community members and activists, we found that participants focused on three key thematic elements when discussing their experience of engaging with a community mobilization intervention. Participants noted that behaviour change for gender equity and HIV risk reduction was facilitated through developing consciousness of and skills in communication, respect and empathy.

In this paper, rather than focusing on behaviour change outcomes (see [31] for outcomes), we were interested in understanding the process that participants went through and relating this to the domains of community mobilization that we have previously developed. While we have discussed ‘change’ in this paper as a result of participation in the community mobilization intervention, this should not be viewed as a clear timeline of incremental changes from the first interview to the last. During the interviews, participants meandered through discussions of their involvement in the intervention and discussed the same issues at multiple time points. Participants spoke about a ‘before’ and ‘after’ the intervention rather than reflecting on incremental changes aligned to the timing of the data collection.

The three key thematic elements reflect critical components of the mobilization domains on which the intervention was premised [27]. Communication is a significant component of the development of critical consciousness, while empathy and respect feed into the development of shared concerns and social cohesion. This mutual and shared view of common interests maps onto the mobilization domain of building community cohesion, though participants seemed to mostly internalize this aspect in direct relation to their families before widening empathetic responses to the community more broadly. We have previously shown in this community that among the domains, critical consciousness seems to be the first ‘achievable’ domain followed by social cohesion [40]. The data presented here confirm our previous findings that these early domains in community mobilization are being achieved, while there is less evidence to suggest the ‘more difficult’ aspects of mobilization are underway.

Process evaluations of other gender-focused interventions have noted the development of many of the same elements. In a longitudinal qualitative case study of men involved in the Stepping Stones programme in KwaZulu-Natal, Gibbs et al. [41] noted increased communication alongside reduced intimate partner violence and avoiding conflict. Similarly, couples engaged in SASA!, a community mobilization intervention aimed at reducing HIV and violence against women in Uganda, increased ‘relational resources’ such as communication and self-regulation, but also showed shifts in relationship dynamics, including increased respect [42]. Our findings on the role of communication illustrate how OMC participants used new skills to reduce the use of violence in their families more broadly than with intimate partners, but that improved communication about taboo and stigmatized issues relating to sexual behaviour and gender relationships also emerged as a process of change in the broader community.

At a community level, felt and demonstrated respect for others was associated with the community mobilizing domains of shared concerns–generating a sense that issues, such as violence, HIV prevention, and even domestic work, are collective and need to be addressed collectively—as well as the development of social cohesion. Although respect was discussed as a positive impact of OMC on the community as a whole, respect was also the concept around which participants expressed the most tensions, implying the zero-sum vision of power, in which female gains in power can only eventuate if men lose power [43]. Participants’ discussions of respect in the context of gender equality showed that changes in attitudes and behaviour were not always easily achieved. Previous research in gender equitable interventions has shown an ‘ongoing tension between the rights-based discourse of gender equality and local cultural discourses of masculinity and social power’ [44], with a sense of male gendered power loss being a common reaction to increased gender equity [45–48]. It seemed that among our participants this tension was particularly felt within the domestic sphere, as it was here that men deployed respect as a patriarchal tactic by specifically articulating the need for women to continue to respect their male partners, despite broader social changes. Given the significant advances in women’s rights entrenched in the South African constitution [49], the domestic sphere possibly represents the final frontier in which men might grapple with maintaining their power over women. Respect in the context of this intervention was often actioned through increased male sharing of domestic responsibilities. While this represents a limited way in which gender inequalities need to be addressed in South Africa, it does offer an opportunity to open dialogue about social constructions of gendered roles and may be the start to initiating more complex conversations about other gendered issues. Such dialogues are the start of a process of changing social environments and take time to achieve [50].

The importance of communication emerging from these data echoes previously identified intervention best practices that have consistently documented the need for communication, and indeed have noted that communication from national campaigns as well as interpersonal communication with partners or family is associated with reduced HIV risk behaviours [51–56]. Our community mobilization framework included a focus on the need for dialogue to generate critical consciousness and ‘shared concerns’ in the community in order for community mobilization to be effective. The majority of participants noted that communication was a skill they perceived to have learned from OMC and that it had improved the quality of their own and their families’ lives. At the individual level, it seemed that learning to communicate changed relationship dynamics and created space in which topics such as gender and HIV might be discussed. Further, consistent with the critical consciousness domain, the type of communication being discussed by our research participants went beyond individual level communication. Participants reported that communities were talking about HIV more often and that issues relating to gender inequalities, such as the need for men to share domestic work, had gained greater prominence in community discussions and dialogue in private and public spheres. Participants also cited the way that opening lines of communication brought new understanding and respect and empathy for others’ experiences.

We have found little in the literature about the role of empathy in accounting for changes in gendered and HIV risk behaviours, as empathy is not often theorized at the community level. However, empathy is a building block and individual-level analogue of one of our framework domains—social cohesion. Cohesion, or a sense that there is a ‘glue’ that holds people together in public health has been at the centre of community empowerment projects, most successfully with populations such as sex workers where collective identities help promote empowerment towards community health [57, 58]. Collective efficacy theorists hypothesize that for communities to tackle social problems, some level of baseline social cohesion, based on working trust and mutual expectation to intervene for shared interests, should exist among community members [59, 60]. While building empathy was not a purposeful focus of the OMC work, building shared interests and trust as well as visioning others in the communities as allies was a focus—and seems to have translated at a basic, individual level more so than on a community level in this data. Empathy was discussed in terms of gender equality (men) and in terms of changing violent behaviour towards children (women). Specifically, men discussed beginning to understand the unequal burden of domestic responsibilities on women and how this understanding encouraged them to participate in housework and childcare. Such changes are significant, as pervasive attitudes towards gender division of labour in the domestic and care economies limit women’s participation in the economy and educational attainment, and heighten gender inequalities. There are significant short- and long-term advantages to men and children for greater male involvement in the care economy [61].

### Limitations

We attempted to maintain a distance between the intervention and the qualitative data collection teams to encourage participants to feel that they could openly discuss problems and failures of the intervention. However, there remains the potential for participants to have exaggerated the impact of the intervention due to a desire for the intervention, and their role in it, to be viewed positively. We also sampled participants from villages that were perceived by OMC staff to be both challenging and easy to work with, to ensure that we did not only present perspectives from villages in which the intervention was well-accepted. One of the challenges of interventions aimed at structural issues such as gender is that change is slow and that the duration of the data collection process may not have allowed participants to reflect on gradual processes that may extend well beyond study duration.

The qualitative interviewers in this study were all female and interviewed both male and female participants. While the impact of gender incongruence on qualitative data has often been negatively portrayed, there is some evidence that women interviewing men is less problematic than the reverse [62]. We acknowledge this potential limitation.

## Conclusions

This study focused on participant and mobiliser experiences of a community mobilization intervention that encouraged men and women to challenge HIV risk behaviours and gender norms in the context of high HIV prevalence in a rural South African community. The results of this longitudinal qualitative study demonstrate that the primacy given to communication, empathy and respect by participants represents initial steps in achieving the essential domains of community mobilization necessary for behavioural change. They should also be viewed as essential components of the initial steps towards achieving intervention outcomes of reducing gender inequalities and HIV risks.

## Supporting information

S1 Appendix Community member interview guide. (DOCX)

S2 Appendix Community mobilizer interview guide. (DOCX)

S3 Appendix Community action team FGD guide. (DOCX)

S4 Appendix COREQ checklist. (PDF)
