# Supplementary material for: Process elements contributing to community mobilization for HIV risk reduction and gender equality in rural South Africa
Source: PLoS One. 2019 Dec 2;14(12):e0225694. doi: 10.1371/journal.pone.0225694 (PMC6886772; doi:10.1371/journal.pone.0225694)
Supplement: S1 Appendix — (DOCX) [file pone.0225694.s001.docx]

**Effect of Community Mobilization on HIV prevention for young South African women**

**Interview topic guide – Community members**

**Background information** -- BASELINE INTERVIEW ONLY [Review previous transcript to make sure that you have this information. Only ask Q1-5 again if the information is missing from the first interview]

1. To start our discussion for the first time, can you tell me a little about yourself?
   - Age, profession, employment
   - Family structure
   - Which village you live in
   - Origins and time living in this community
2. What community structures are you involved in? (probe: political, church, volunteer, sports, school, cultural, wo/man’s, savings)
   - Explain what each group does
   - What impact does each group have on the community
3. What other groups or community organizations do you have in this community? What do they do?
4. Is this community a good or a bad place to live? Can you explain why you think that?
5. Tell me about the leaders in this community.
   - What makes them leaders?
   - Effectiveness
   - Trust

**HIV prevention in communities**

1. Are people concerned with HIV in your community? Why or why not?

- What is the community concerned about that is related to HIV/AIDS?

1. How has the community tried to deal with HIV/AIDS or its impact? What has the community response been? Probe: programs / social services / community support.
2. Tell me about HIV prevention programmes in this community. How did you learn about these programmes? (If none: How do people in this community learn about HIV?)
3. What is the impact of HIV prevention programmes in this community?

**Genders issues in communities**

1. What are the roles of women in your community? What are the roles of men? (In terms of family, work, decision making). Have these roles changed further since we last spoke?
2. What are your views about gender equality?
3. Is gender-based violence occurring now and if yes, why? (Probe: Is it justifiable?)
4. Is gender-based violence (or violence against women) a concern in your community? Why or why not?
5. What about other forms of violence, like violence against children? (Probe: who are the perpetrators? How much of a concern is this for the community?)
6. Tell me about the gender equality organisations or campaigns in your community. (Probe: Organisation against gender violence)

- What is the impact of these organisations in promoting gender equality in this community?
- [Any changes since we last spoke]?

1. What are neighbours and the community expected to do in this community when they know about a problem (for example: gender violence)?
   - Do people get involved or do they choose not to? Why do they make this choice?
2. What do you think is a relationship between HIV and gender roles or norms?

**“One Man Can” campaign knowledge**

1. How well known is the “One Man Can” campaign in this village? [Probe for approx. % who know of campaign]
   - [More known since we last spoke? The same? Profile declining]?
2. What are people in your community currently saying about “One Man Can”?
   - Mostly positive or mostly negative?
   - Who is talking about it?
   - When and how did you first hear about it?
3. *[follow up only]* Have you learned something new about “One Man Can” since the last time we talked?

**“One Man Can” participation and evaluation**

1. Have you personally been involved in any of the “One Man Can” activities [since we last spoke]? What?
2. Tell me about your experience of participation (one-to-one; workshop; outreach [type]) *Ask probe questions of all activities that participant reports*
   - Describe the level of participation in this activity (number of participants but also degree of engagement with the activity)
   - Who were the people that were involved in the activity (gender, age, status in community, formal structures or informal grouping)
   - What was the goal of the activity? Do you think that this was achieved?
3. Has your experience of “One Man Can” been enjoyable? Why or why not?
4. Who is getting involved? Who isn’t? (probe men, women, youth) Why is this the case?
5. *[follow up only]* Since we last spoke, do you think that there has been any meaningful change in terms of community discussion or action around gender norms and HIV?
   - What has changed? How is this change related to OMC?
   - If no – why? What are the barriers to changing gender norms or community interest in HIV?
   - Do you think that OMC has made an impact on violence against children?
6. *[follow up only]* Since we last spoke, what has changed for you? Has it changed the way you interact w/ your community? Has it changed the way your community interact with one another? If yes, how? why?
   - Have you been involved with community organizing for change? Is your community open to change? Why / why not?
7. Has it changed the way you behave? If yes, how? Why? (probe: in romantic relationships? At home with family? With kids (esp violence)? Clinic attendance? HIV testing?)
   - Are there any choices you’ve made about the way you live that were affected by your participation?
