# Supplementary material for: Process elements contributing to community mobilization for HIV risk reduction and gender equality in rural South Africa
Source: PLoS One. 2019 Dec 2;14(12):e0225694. doi: 10.1371/journal.pone.0225694 (PMC6886772; doi:10.1371/journal.pone.0225694)
Supplement: S2 Appendix — (DOCX) [file pone.0225694.s002.docx]

**Effect of Community Mobilization on HIV prevention for young South African women**

**Interview topic guide – Community Mobilizers**

**Background** – BASELINE INTERVIEW ONLY [Before round 2 interviews please check previous transcript to ensure that we have this information. If not, please ask these questions again]

1. Tell me a little bit about yourself
   - Family, interests, involvement in One Man Can (OMC), role etc.
2. Why were you interested in getting involved in OMC as a mobilizer?
3. What did you think about gender equality before you were trained as an OMC mobiliser?
4. How did your family shape your views of gender and gender equity?

**Community Engagement**

1. How well do you think community members are engaging in the “One Man Can” activities [since we last spoke]?
2. Which topics/activities are the easiest to get people to engage with? Why?
3. Which topics/activities are the most difficult to get people to engage with? Why?
4. What are currently the main barriers to engagement?
   - Different or the same across all villages?
5. Who are currently the main people engaging with the activities?
   - Probe for age, gender, formal structure membership
   - Is this changing?
6. What is it that makes some people engage with the activities and others not? Any strongly emerging patterns?
   - Individual characteristics or village characteristics?
7. How has engagement changed [**since we last spoke**]?
   - Number of people
   - Type of people
   - Level of participation
   - Same or different across villages? Give examples
8. Why do you think that the level of engagement is changing?
9. What are the things that have most facilitated community engagement with the activities recently?
   - Different or the same across all villages?
10. How engaged has the Community Action Team in *[insert name of village]* been [since we last spoke]?
    - Reasons for level of engagement?
    - Better or worse than other communities?

**Perceived effect of Intervention**

1. What impact do you feel the “One Man Can” activities have had in communities [**since we last spoke**]?
   - Evidence for this?
2. Can you tell me about changes that have occurred in your community [since we last spoke] that can be attributed to OMC? (**Probe**: changes in terms of gender equity; violence against children; engagement in HIV issues). Who are the main perpetrators of violence against children? Has this changed?

- ***If no evidence of change****:* Why do you think there has been no change?

(**Probe**: Intensity, participation, format, content, leadership support)

1. Can you give me some specific examples of how OMC activities have influenced change?
2. What do you think can be done or changed/improved to make a larger impact?

**Personal Change**

1. How have your views on the role of women and men in our communities changed [since we last spoke]?

- **Probe:** Men washing dishes and childcare, women working, gender violence, violence against children, HIV, etc.
- **Probe:** Are you more involved in working with the community on problems outside of OMC’s focus? Is this a change or how it always was? Why do people choose to get involved or not?

1. What do like most/least about being a community mobilizer?
2. Of the OMC messages you teach community members, which do you agree with the most? Disagree with or are unsure about? [*Remind mobilisers this is anonymous and has no bearing on employment status*]
3. Which OMC messages have been difficult to communicate to men? Women?
4. Tell me about friends, family members, or community members who may not support your participation in OMC? Are there individuals who are especially supportive? Please describe.
5. In your own family life, or relationships, tell me about how you have made changes based on what you’ve learned from OMC, [since we last spoke]?
   - **Probe**: household chores, treatment of female family members, violence, condom use or other HIV prevention behaviors
   - Have these changes been easy or difficult? What has it been like for you?
   - What has been the easiest change? Hardest?
6. Can you describe a time in the past few months when you realized you acted in a way that was counter to the OMC ideas about gender equality?

- How do you reconcile this with your involvement in OMC? What do you think that the community would think about this?

1. Tell me about anything else that has changed in your knowledge; viewpoints; attitudes [since we last spoke]?
2. From your point of view, what was this experience like for OMC participants? What praise/criticism have you heard about OMC [since we last spoke]?
