# Supplementary material for: Process elements contributing to community mobilization for HIV risk reduction and gender equality in rural South Africa
Source: PLoS One. 2019 Dec 2;14(12):e0225694. doi: 10.1371/journal.pone.0225694 (PMC6886772; doi:10.1371/journal.pone.0225694)
Supplement: S3 Appendix — (DOCX) [file pone.0225694.s003.docx]

**Effect of Community Mobilization on HIV prevention for young South African women**

**FGD topic guide – Community Action team**

**Motivation to and Experience of CAT**

1. What are the main reasons that you chose to be part of this CAT? [first interview only]
   - Probe for personal stories if possible [background information that shows why they participate in this type of activity]
2. Tell me about your experience of participating in OMC workshops
   - Favourite component of workshop
   - Least favourite component of workshop
3. What do you think was the most valuable skill you learned in the workshops?
   - Why is this valuable?
4. What do you think was the least valuable skill you learned in the workshops?
5. How could OMC workshops be improved in the future?
   - Content, duration, focus, balance of skills vs activity training, location, timing
6. Tell me about your experience of running OMC activities
   - Balance of activities – which do they do most?
   - Activity preference
   - Barriers and facilitators
   - Monitoring
   - Feeling of competence
   - Additional training needs
7. How appropriate do you feel the OMC messages are for this community? Why?

**Community Engagement**

1. How well do you think community members are engaging in the “One Man Can” activities?
2. Which activities are the easiest to get people to engage with? Why?
3. Which activities are the most difficult to get people to engage with? Why?
4. What are the main barriers to engagement?
5. What are the things that have most facilitated community engagement with the activities?
6. Who are the main people engaging with the activities?
   - Probe for age, gender, formal structure membership
   - Is this changing?
7. What is it that makes some people engage with the activities and others not? Any strongly emerging patterns? Individual characteristics or village characteristics?

How has engagement changed [**since we last spoke**]?

- - Number of people
  - Type of people (age, gender, formal structure membership)
  - Level of participation

1. Why do you think that the level of engagement is changing?
2. How is your CAT finding working with leadership? Probe for particular challenges

**Perceived effect of Intervention**

1. What impact do you feel the “One Man Can” activities have had in communities [since we last spoke]?
   - Evidence for this?
2. In which focus area of the intervention has the impact occurred? [HIV or gender]
   - Why this one?
   - Why not the others?
   - Has there been any change in violence against children?
   - Have there been changes in people working together to solve problems outside of OMC focus on HIV and gender?
3. *If no evidence of change:* Why do you think there has been no change?
   - Intensity, participation, format, content, leadership support
4. Can you give me some specific examples of how OMC activities have influenced change?

**CAT level change**

1. How has the CAT evolved since the last time you spoke with us?
   - Membership
   - Attitudes, committment
   - Methods
